# Optical transient grating pumped X-ray diffraction microscopy for studying mesoscale structural dynamics

**DOI:** 10.1038/s41598-021-98741-y

**Published:** 2021-09-29

**Authors:** Travis D. Frazer, Yi Zhu, Zhonghou Cai, Donald A. Walko, Carolina Adamo, Darrell G. Schlom, Eric E. Fullerton, Paul G. Evans, Stephan O. Hruszkewycz, Yue Cao, Haidan Wen

**Affiliations:** 1 Materials Science Division, Argonne National Laboratory, Lemont, IL 60439 USA; 2 Advanced Photon Source, Argonne National Laboratory, Lemont, IL 60439 USA; 3 Department of Materials Science and Engineering, Cornell University, Ithaca, NY 14853 USA; 4 Kavli Institute at Cornell for Nanoscale Science, Ithaca, NY 14853 USA; 5 Leibniz-Institut Für Kristallzüchtung, Max-Born-Str. 2, 12489 Berlin, Germany; 6 Center for Memory and Recording Research, University of California San Diego, La Jolla, CA 92903 USA; 7 Department of Materials Science and Engineering, University of Wisconsin−Madison, Madison, WI 53706 USA

**Keywords:** Characterization and analytical techniques, Electronic devices, X-rays, Ferroelectrics and multiferroics

## Abstract

A fundamental understanding of materials’ structural dynamics, with fine spatial and temporal control, underpins future developments in electronic and quantum materials. Here, we introduce an optical transient grating pump and focused X-ray diffraction probe technique (TGXD) to examine the structural evolution of materials excited by modulated light with a precisely controlled spatial profile. This method adds spatial resolution and direct structural sensitivity to the established utility of a sinusoidal transient-grating excitation. We demonstrate TGXD using two thin-film samples: epitaxial BiFeO_3_, which exhibits a photoinduced strain (structural grating) with an amplitude proportional to the optical fluence, and FeRh, which undergoes a magnetostructural phase transformation. In BiFeO_3_, structural relaxation is location independent, and the strain persists on the order of microseconds, consistent with the optical excitation of long-lived charge carriers. The strain profile of the structural grating in FeRh, in comparison, deviates from the sinusoidal excitation and exhibits both higher-order spatial frequencies and a location-dependent relaxation. The focused X-ray probe provides spatial resolution within the engineered optical excitation profile, resolving the spatiotemporal flow of heat through FeRh locally heated above the phase transition temperature. TGXD successfully characterizes mesoscopic energy transport in functional materials without relying on a specific transport model.

## Introduction

The nanoscale, nonequilibrium behavior of materials underpins a host of phenomena, from ultrafast optical activation of functional electronic and magnetic properties^1^, to laser-based additive manufacturing^2^, to solid–solid phase transformations relevant to neuromorphic computing^3^. Simultaneously measuring the ultrafast, nanoscale dynamics of materials promises to advance such fields. Several characterization techniques provide either spatial or temporal resolution, but, as we demonstrate, unique insight can be gained by having both spatial and temporal resolution simultaneously. Here, we apply high spatial resolution and direct lattice sensitivity to the transient grating (TG) technique by employing a focused X-ray probe to resolve the evolution of the excited structural grating in real space.

Optical TG has been widely applied in studying nonequilibrium material dynamics. TG techniques employ a spatially structured intensity pattern, generated via optical interference, with a sinusoidal spatial dependence. Absorption of the optical energy from the interference pattern excites a response in the sample with a commensurate spatial profile. The grating induced in the material takes the form of periodic regions of varying temperature, carrier density, or magnetic moment^4^. In optical TG methods, an optical probe measures the time dependence of the material response via diffraction from the grating. Optically probed TGs are useful in characterizing, for example, the thermal and elastic properties of thin films^5,6^ and the electronic behavior of superconducting materials^7^. Optical probes, however, have spatial resolution limited to the scale of the optical wavelength and do not provide direct structural information. In order to provide structural information, hybrid X-ray-optical TG techniques have been introduced, where either the excitation^8^ or the probe^9,10^ employ X-ray pulses with a wavelength far shorter than that of optical pulses. Hard X-ray probes are directly sensitive to the lattice spacing, which is critical for characterizing functional materials like ferroics, where the lattice interacts strongly with the electron charge and spin degrees of freedom^11^. Previous X-ray studies of TG phenomena have recorded only the intensity at the first-order diffraction maximum, the lowest diffraction angle, and thus provide only properties that are averaged over the full area of spatially modulated excitation. The first-order X-ray diffraction method of probing overlooks spatial variations arising from nonlinear responses, e.g., phase transitions or non-diffusive transport, both of which are common at the nanoscale^12,13^. New experimental methods, as we demonstrate here, provide direct lattice sensitivity and simultaneously high spatial and temporal resolution, which help provide insights into important dynamical processes such as phase transformations and thermal transport at the mesoscale.

**Figure 1:**
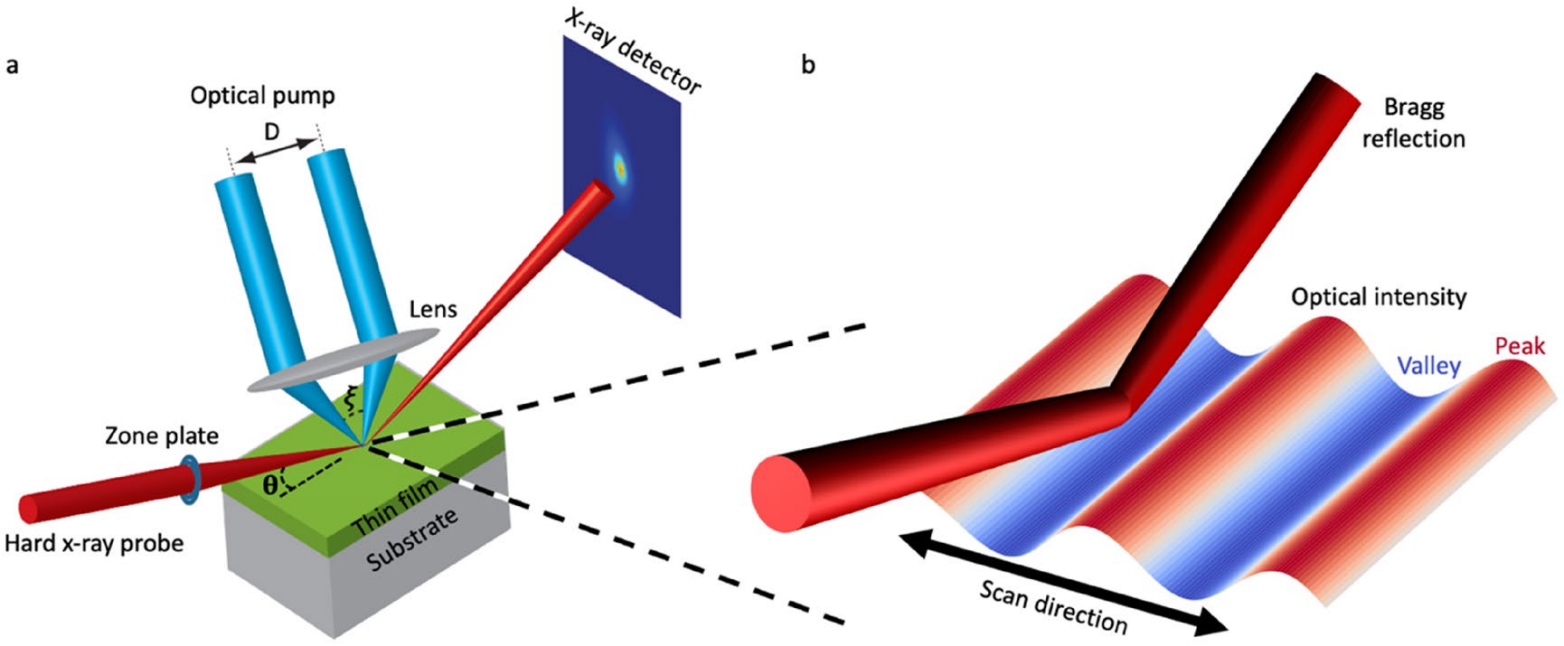
Experimental arrangement for the transient grating pump and focused X-ray diffraction probe (TGXD) technique. (**a**) Two synchronized optical pulses with crossing angle x generate a transient grating on the sample. After a controlled delay, the focused X-ray probe diffracts from the film and is collected by a two-dimensional X-ray detector. (**b**) Zoom-in view of the excited region. Scanning the TG relative to the focused X-ray beam spatially resolves the evolution of the structural grating.

The optical transient grating pump, synchrotron X-ray micro-diffraction probe (TGXD) method reported here uses a focused X-ray beam to locally probe within the TG period, rather than area-averaged diffraction from several TG periods (see Fig. 1). The X-ray focal spot, with a full-width-at-half-maximum of 270 nm measured by the intensity profile, provides greatly improved spatial resolution, and extends beyond previous reports of locally probed TG, which were limited to optical wavelengths^14,15^. We generate TG excitation by crossing two 355 nm wavelength laser beams on the sample surface (see “Methods” section). The tunable crossing angle controls the periodicity of the resulting interference pattern. We then probe the sample response by recording the diffracted X-ray intensity near the Bragg condition as a function of the pump-probe delay time, *t*, the relative spatial position of the TG pattern and the focused probe, and the X-ray incident angle, *θ*. The TGXD method provides transient excitation with a high spatial gradient and precise structural measurements of the response and dynamics.

To demonstrate the TGXD approach, this report studies two materials systems exhibiting significant differences in their response to optical excitation. The first system is a multiferroic BiFeO_3_ (BFO) thin film, in which the photoinduced strain was proportional to the optical intensity. The induced structural grating in BFO followed the intensity profile of the optical pump and relaxed at microsecond timescales, similar to the carrier recombination time^16^. The characterization of BFO helps to estimate the systematic errors of the TGXD method because the optically induced strain grating in BFO is known to be strictly sinusoidal. The second system is a FeRh thin film, which exhibited a nonlinear response due to a photoinduced structural phase transition^17^. The transient recovery from the ferromagnetic (FM) to the antiferromagnetic (AFM) phase of FeRh occurred with a non-sinusoidal spatial profile along the TG, deviating from the intensity profile of the optical pump. The non-sinusoidal real-space profile would not have been captured by the previous first-order diffraction measurements of TG excitations. The TGXD technique allowed thermal transport to be tracked for both phases during the first-order transition, via direct structural characterization of the engineered heterogeneous phase distribution evolving at the mesoscale.

## Results

### Linear response in BiFeO_3_

BFO is a multiferroic with a distorted perovskite crystal structure. The photovoltaic, piezoelectric, and magnetoelectric functionalities of BFO are of widespread interest^18–20^. BFO develops substantial structural distortion upon illumination by above-band-gap light due to the separation of charge carriers that screen the depolarization field^21,22^.

**Figure 2:**
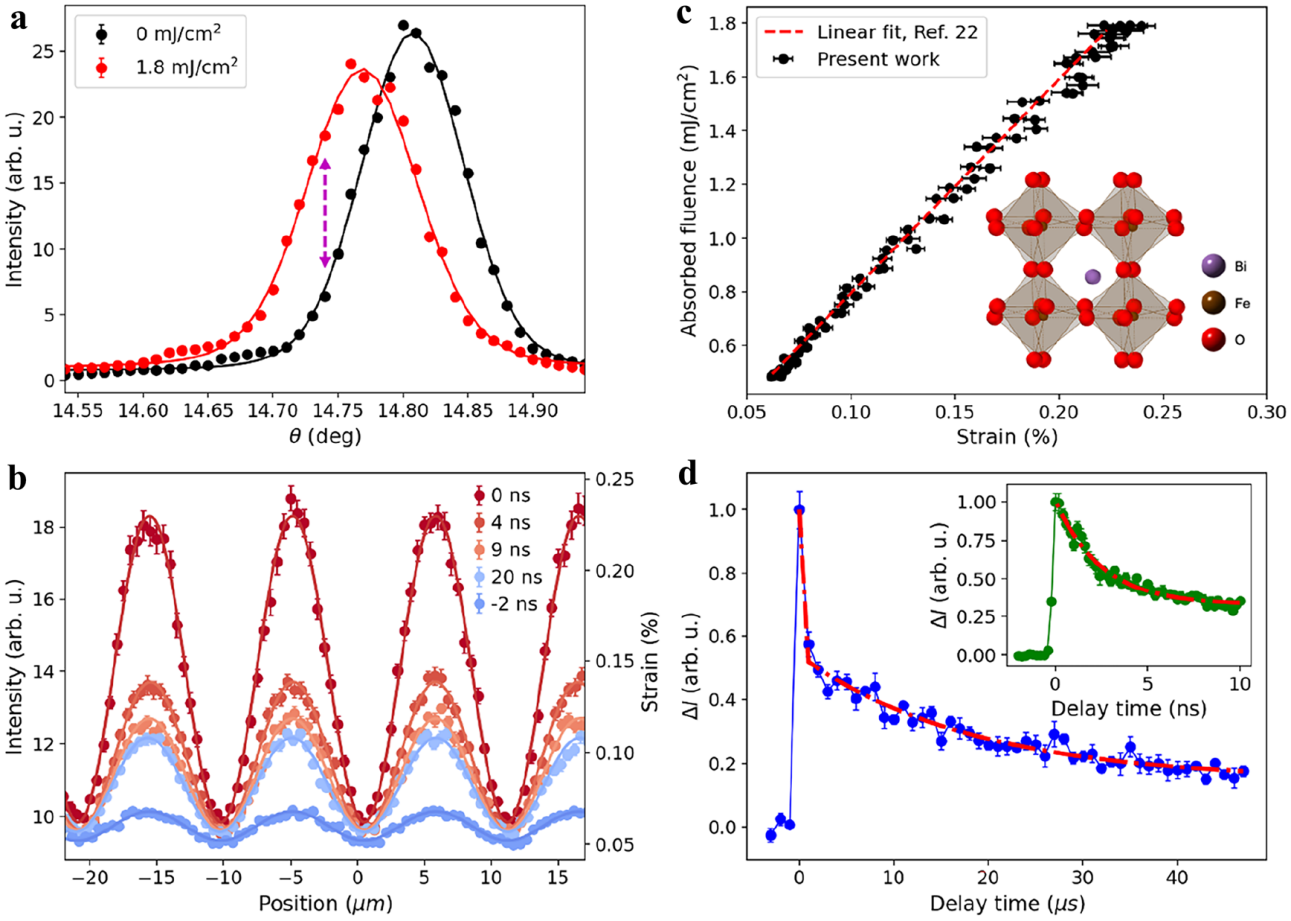
Linear strain dynamics in BFO. (**a**) Shift in the 002 pseudocubic Bragg peak at the TG peak position and *t* = 0 ns for zero and maximum laser fluence. The dotted arrow indicates the fixed value of *θ* used for the measurements shown in (**b,d**). (**b**) X-ray intensity and corresponding strain as a function of TG position for several delay times. Solid lines are sinusoidal fits. (**c**) The estimated fluence as a function of the measured strain at 0 ns extracted from (**b**), based on the linear relationship (dashed line) from Ref.^22^. Inset: Perovskite BFO structure showing Bi (purple), O (red), and Fe (brown) atoms. (**d**) Changes in X-ray intensity at the TG peak as a function of time, fit with a bi-exponential decay (red dotted line) with time constants 2.5 ns and 18 μs. Inset: higher resolution delay scan in the first 10 ns. Error bars are described in “Methods”.

We characterized the structural response by measuring X-ray diffraction signatures associated with the distortion of the BFO lattice after excitation by a 54 kHz repetition rate laser. Symmetric *θ*–2*θ* X-ray diffraction scans revealed the shift of the 002 Bragg peak, as shown in Fig. 2a for zero laser fluence and the maximum fluence probed in these experiments (approximately 1.8 mJ/cm^2^ absorbed fluence). The maximum observed shift in the Bragg angle was 0.04°, measured at the location of maximum TG excitation fluence and the pump-probe delay time *t* = 0, as defined in “Methods” section, with 111 mW laser power. The shift in the Bragg angle corresponds to a strain of 0.25%.

For sufficiently small shifts, the strain is proportional to the change in the X-ray intensity measured at a fixed value of *θ* on the low-angle shoulder of the Bragg peak. We derived the coefficient of proportionality between the change in intensity and the strain from fits of Voigt functions to the *θ*–2*θ* scans (see Supplementary Information). For *θ* = 14.74°, indicated by the arrow in Fig. 2a, the value of the coefficient is 2 × 10^–5^ strain per 1% change in X-ray intensity. This relationship allowed temporal and spatial scans to be acquired at a fixed value of *θ* for more rapid data acquisition.

As shown in Fig. 2b, the strain in BFO followed the sinusoidal profile of the TG excitation at all delay times. Because the local laser fluence varied across the TG profile, the sinusoidal pattern indicates that the strain is proportional to the pump fluence, consistent with past work^22^. We determine the range of fluences along the TG profile measured at *t* = 0 in Fig. 2b by converting the measured strain (black points in Fig. 2c) into fluence, using the linear relationship (dashed line, Fig. 2c) between absorbed fluence and strain from Ref.^22^.

The spatial scans at different fixed delay times show that the strain grating persisted at negative delay times, where the X-ray pulse arrived before the laser pulse. The strain grating thus persisted for the entire interval between optical pulses, indicating that the structural recovery was not completed in the 18 μs interval between adjacent TG pulses at the 54 kHz repetition rate. To probe the full relaxation of the optically induced structural perturbation in BFO, we next switched to a low laser repetition rate of 5 kHz, that is, a 200 μs pulse-pulse interval.

With the extended pulse-pulse interval, we measured the evolution of the sample at a position of peak TG intensity. There was no persistent signal at negative delays, confirming the full relaxation was captured within this interval. As shown in Fig. 2d, there were two well-separated time scales in the BFO response, t_1_ = 2.5 ± 0.2 ns and t_2_ = 18 ± 2 μs (see Supplementary Information). We determined the timescales from a biexponential fit that also included a vertical offset, indicating that there was a third, longer, decay time that was approximately constant over our measured window. The 2.5 ns time constant agrees well with both reported strain relaxation measurements^22^ and the fast component of carrier recombination times^23^. The 18 μs time constant for the second stage is longer than the expected time for the temperature of the BFO thin film to be reduced to a factor of 1/e from its initial value by conduction, which is tens of ns^22^. The 18 µs time is, however, within a factor of two of the lifetime of photoexcited carriers measured by photoluminescence (9.5 μs)^16^ and photocurrent (35 μs)^24^. The observation of this time constant is consistent with the carrier screening mechanism responsible for strain generation in BFO^22^.

The TGXD study of BFO also serves as a benchmark to determine the confidence interval of measuring a sinusoidal strain profile, given the known linear response of BFO. The results in Fig. 2b agree with pure sinusoids within the error bars, which represent the systematic error in the experiment due to factors such as position jitter, angular stability, and pump fluence fluctuations. Thus, for measurements on other materials, non-sinusoidal deviations larger than the error bars can be attributed to the sample response rather than experimental uncertainty. This is important for studying nonlinear structural responses, such as the phase transition involved in the next case study of FeRh.

### Nonlinear response in FeRh

**Figure 3:**
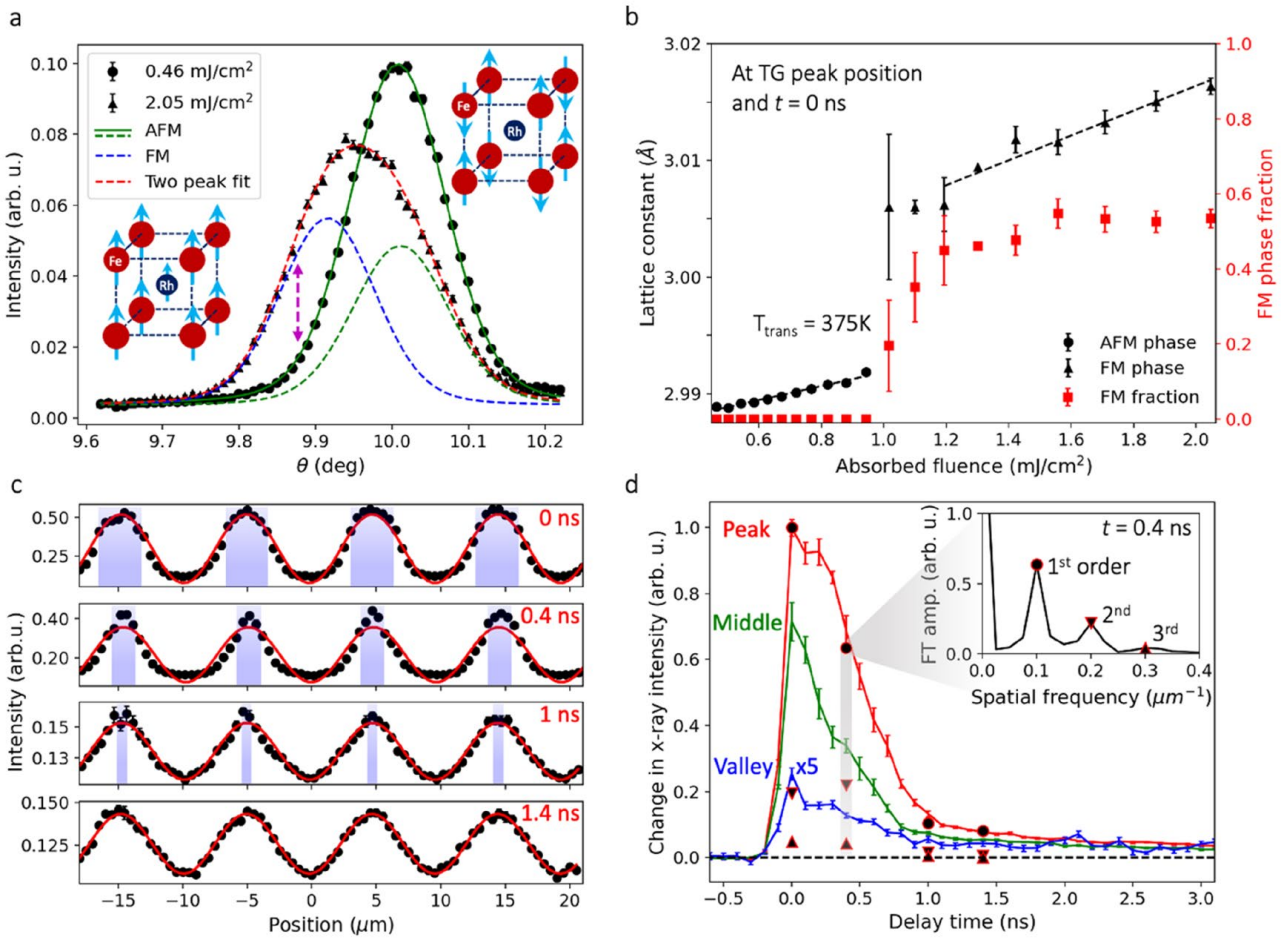
Nonlinear structural dynamics in FeRh. (**a**) 001 Bragg peak measured at the TG peak at *t* = 0. The peak at low excitation fluences (circles) is in the AFM phase (solid green line fit). At higher fluence (triangles) there is a mixture of AFM and FM phases. Dashed lines fit the contributions of the AFM (green) and FM (blue) phases to the high-fluence curve (red dashed line total). The vertical arrow indicates the value of *θ* for the measurements shown in (**c,d**). The insets are diagrams of the cubic FeRh lattice showing Fe (red) and Rh (blue) atoms, and the directions of the magnetic moments (arrows). (**b**) Lattice constant and FM phase fraction as a function of absorbed laser fluence. Dotted lines are separate linear fits to the AFM and FM peak centers. (**c**) Diffracted intensity as a function of TG position and delay time with 1.4 mJ/cm^2^ peak absorbed fluence. Lines are sinusoidal fits. The shading indicates the approximate locations of regions in which the FM phase occurred. (**d**) Non-exponential decay in X-ray diffraction intensity at various TG positions. The curve for the minimum TG excitation has been multiplied by a factor of 5. The points show the normalized change in the amplitude of the first- (circles), second- (downward pointing triangles), and third- (upward pointing triangles) order Fourier components of the spatial profiles in (**c**). The inset shows the amplitude of the Fourier transform of the TG profile at 0.4 ns. Error bars are described in “Methods” section.

FeRh exhibits a first-order magnetostructural phase transition upon optical excitation^17,25^. This AFM-to-FM phase transition also occurs in steady state at T_trans_ = 375 K, with a concurrent lattice expansion^26,27^. As shown in Fig. 3a, Bragg reflections at a fluence near the phase transition threshold exhibited contributions from both the AFM and FM phases, which have different lattice parameters. The observation of phase coexistence within the probed volume at *t* = 0 resulted partly from the limited penetration depth (~ 10 nm) of the pump beam, which heated primarily the region near the film surface. The phase coexistence at *t* = 0 is also consistent with the ~ 200 ps saturation time for the phase transition at these fluences^17^.

To observe the evolution of this transient grating of mixed phases at each TG position, we used both qualitative measurements at fixed *θ* (arrow in Fig. 3a), and quantitative measurements of full *θ*–2*θ* scans. First, *θ*–2*θ* scans as a function of pump fluence at the TG peak position and *t* = 0 identified the threshold fluence for the phase transition. Above the threshold fluence, the Bragg peak changed shape due to the contribution of the FM phase. Voigt function fits to the FM and AFM contributions to the Bragg peak determined the relative fraction of the FM phase, as shown in Fig. 3b. Below this threshold fluence, we fit a single Voigt function to the Bragg peak to track the AFM lattice expansion. After finding the threshold fluence to be ~ 1 mJ/cm^2^, we next selected a TG peak fluence of 1.4 mJ/cm^2^ for subsequent fixed-*θ* measurements.

Fixed-*θ* measurements along the TG show how the fluence of 1.4 mJ/cm^2^ triggered the AFM-FM transition only in the vicinity of the peak TG positions (see Fig. 3c). The local phase transitions produced a position-dependent structural response that deviated from the sinusoidal excitation profile. We estimate the approximate regions along the TG profile that were in the FM phase with the shaded areas in Fig. 3c. The minima of the curves in Fig. 3c have been aligned by applying a long-term drift correction proportional to the elapsed measurement time.

The Fourier transform of the measured spatial profiles determines how the TG response in FeRh deviated from a purely sinusoidal profile. The resulting spatial frequency spectrum shows Fourier components up to the third order were present for the first nanosecond of cooling, as shown in the inset of Fig. 3d. These features would not be detected by area-averaged probes recording first-order diffraction directly from the TG pattern.

The fixed-*θ* measurements also tracked how the spatial profile changed during cooling, as the spatial extent of the FM regions shrank. Within the first 0.4 ns, the second-order Fourier component increased, while other orders monotonically decreased, indicating that there was broadening of the valley, corresponding to the growth of AFM regions. Scans with a smaller time step at fixed TG positions show how the decay behavior varied with position, as shown in Fig. 3d. At the TG peak, the intensity is nearly constant for 0.2 ns after excitation, which is a clear signature of the first-order structural phase transition during the cooling of the film^28^.

The results shown in Fig. 3c,d illustrate how the known spatial profile of the TG excitation simplifies the detection of a heterogenous structural response. In comparison, an unstructured optical pump beam would deviate from a gaussian profile due to aberration, optics defects, and alignment errors, while TG provides a strictly sinusoidal spatial modulation without these complications. Thus, the results from TG can be directly compared to models with precisely known excitation profiles.

To extract quantitative structural information such as the film’s phase composition and to track the dissipation of thermal energy in the system, we acquired *θ*–2*θ* scans at each delay time and TG position. We set the TG period at 1.3 μm for these measurements and increased the peak absorbed pump fluence to 5 mJ/cm^2^. This fluence produced complete transitions to the FM phase at the peak TG positions within the first 0.1 ns. Under these conditions, partial transitions occurred at the TG valleys due to the residual optical intensity at the TG minima.

**Figure 4:**
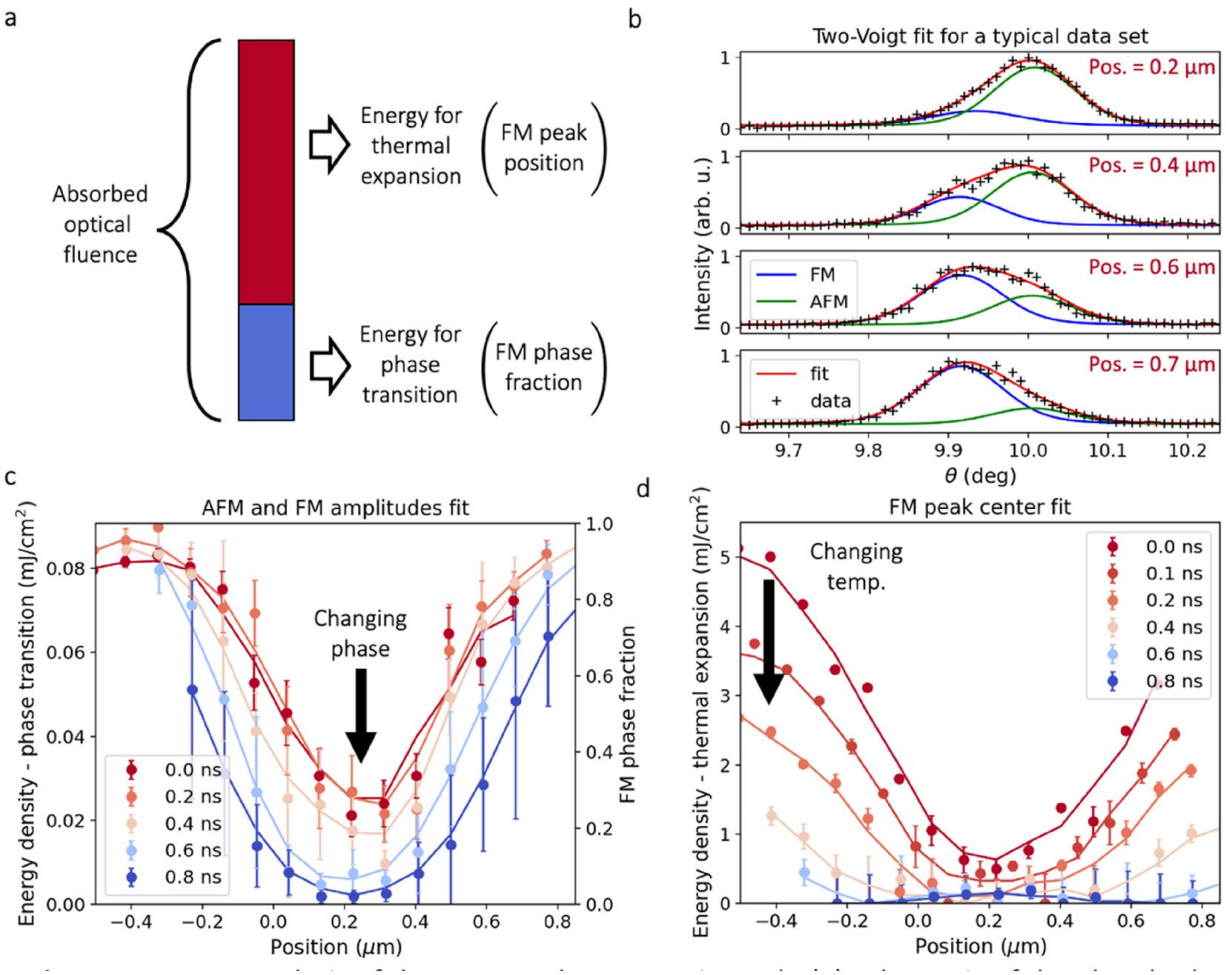
Energy analysis of the structural response in FeRh. (**a**) Schematic of the absorbed energy partitioning. Part of the absorbed fluence triggers the phase transition (tracked by Bragg peak amplitudes), and any excess energy heats the FM phase (tracked by the FM peak center). (**b**) Extraction of the FM phase fraction and FM angular maximum for several positions at *t* = 0. (**c**) FM phase fraction at each position and time, proportional to the energy density locally stored in the phase transition. (**d**) Local energy density remaining in the FM phase heated beyond T_trans_. The data at each subsequent time step in (**c,d**) are shifted by a constant amount such that the minima line up, accounting for the long-term spatial drift during the data collection. Solid lines are three-point smoothed data, accounting for the 270 nm spot size and 90 nm step size. Error bars are described in “Methods” section.

Upon excitation, a fraction of the absorbed optical energy triggered the phase transition, and excess energy heated the FM phase, as shown schematically in Fig. 4a. We fit the resulting mixed-phase Bragg peak with two Voigt functions (Fig. 4b), as described in the Supplementary Information, to study the relaxation process. By analyzing the decomposed AFM and FM Bragg peaks, we disentangled the latent heat of phase transformation from the additional heat in the FM phase above T_trans_. The amplitudes of the FM and AFM peaks determined the FM phase fraction (FM amplitude normalized by the sum of both amplitudes). The angular center of the FM peak measured the thermal expansion of the FM phase (see Fig. 4c,d). Using the specific heat, thermal expansion coefficient, and latent heat from the literature^26,29,30^, we converted the measured FM phase fraction and temperature into local energy densities (see Supplementary Information).

We now interpret the photoexcited response of the FeRh film by comparing the local energy densities for phase transformation and for heating the FM phase. The phase transition saturated within 0.2 ns, as the FM phase fraction stayed unchanged in the first time steps at all TG positions (Fig. 4c). The temperature in the FM phase dropped uniformly (Fig. 4d) over this time. From 0.2 to 0.8 ns, the phase fraction steadily decreased at the TG valley (Fig. 4c) and the FM temperature measured by the FM peak position did not change significantly (Fig. 4d), consistent with a first-order phase transition. At the TG peak, the FeRh fully transitioned to the FM phase (Fig. 4c) and was heated beyond T_trans_. The FeRh uniformly cooled to, but not below, T_trans_ within this time window (Fig. 4d). The steady reduction in thermal energy at all points occurred as heat transferred into the substrate, out of the measured thin film.

X-ray measurements at the TG peak at *t* = 0 show that the total energy density required to induce the observed change was 5.9 mJ/cm^2^, agreeing reasonably with the estimated 5 mJ/cm^2^ absorbed optical fluence (see Supplementary Information)^31–33^. Such results, which are obtained by direct structural measurements without assuming any specific transport model, could be used to test and benchmark advanced transport equations in systems exhibiting non-diffusive thermal transport^12,13^.

## Conclusion

Optical transient grating pumped X-ray diffraction microscopy can provide insight into mesoscopic structural dynamics and energy transport in a wide range of materials. By probing locally across the TG profile, TGXD characterizes transient structural gratings that both follow (e.g., BFO) and deviate from (e.g., FeRh) the optical intensity profile. The measurements extract how the local thermal energy that transferred from the optical pump to the lattice degree of freedom subsequently evolves through the thin film system, including when mixed phases are present. These results provide a detailed view of thermal energy transport without assuming a specific heat transfer model. Instead, with the known specific heat, thermal expansion coefficient, and latent heat, the measured lattice expansion and phase composition directly track the energy in space and time so that the underlying heat transport process, e.g. ballistic or diffusive, can be determined and characterized.

The TGXD method offers a promising avenue for characterizing material systems with novel transport properties and heterogeneous compositions. Although TG-induced thermoelastic responses such as surface acoustic waves were not the focus of our study, TGXD can in principle allow acoustic responses as well as energy transport to be characterized as a function of TG wavevector^5,34^. Beyond structural gratings, we expect similar techniques can measure the energy transport as a result of exciting transient charge and spin gratings. Higher spatial and temporal resolution can be further pursued by the use of advanced X-ray optics and free-electron lasers.

## Methods

In this demonstration of TGXD, a spatially structured optical pump first excited transient dynamics in the sample, then a focused X-ray probe measured the local structural state of the sample. By raster scanning the TG grating with respect to the X-ray beam at various delay times, we measured a spatiotemporal map of the sample response. We excited crystalline thin film samples with a controllable TG pattern generated from ultrafast laser pulses of wavelength λ = 355 nm. The ~ 30 nm thickness of each film was greater than or equal to the optical penetration depth in both FeRh and BFO, ensuring that pump light was absorbed strongly in the films, as described in the SI. To generate a TG pattern, each laser pulse was split into two by a custom interferometer, then focused by a lens to recombine the split pulses with a controlled crossing angle at the sample surface (see Fig. 1, Supplementary Information). The resulting optical interference produced a sinusoidal variation in the optical intensity, *I*:

1 $$\begin{array}{c}I= {I}_{0}+{I}_{0}\;{\mathrm {cos}}\left(\frac{2\pi }{\Lambda }x\right),\end{array}$$

2 $$\begin{array}{c}\Lambda =\frac{\uplambda }{2\mathrm{sin}\left(\upxi /2\right)},\end{array}$$

where *I*_0_ is the total optical intensity entering the system, and ξ is the crossing angle between the two pulses^4^. As apparent in Eq. (2), tuning the crossing angle controlled the resulting TG period.

We synchronized the laser to a sub-multiple of the 6.5 MHz X-ray repetition rate, and electronically controlled the delay of the laser pulses relative to this subset of X-ray pulses. The X-ray detector was gated to only measure the X-ray bunches directly following laser excitation. The zero-delay time, *t* = 0, is defined as the peak in the structural response, corresponding to ~ 50 ps between the centers of the 100 fs-duration laser pulses and the 100 ps X-ray pulses. We used either a 54 kHz laser repetition rate for a better signal-to-noise ratio, or a 5 kHz laser repetition rate to allow the full relaxation of long-lived sample responses. When excitations are long-lived, sequential high repetition rate laser pulses can arrive before the sample fully relaxes, leading to a quasi-steady state offset from true equilibrium at all measured times.

We probed structural dynamics using monochromatic 12 keV X-ray photons focused to a 270 nm full-width-at-half-maximum spot size by a Fresnel zone plate at station 7-ID-C of the Advanced Photon Source (see Supplementary Information). Using a pixel area detector (Pilatus 100 K), we recorded the relevant Bragg diffraction for each sample (BFO 002 or FeRh 001), as a function of delay time, spatial position, and X-ray incident angle, *θ* (see Fig. 1a). The consistency of repeated scans in the same location indicated there was no significant damage due to the focused X-ray beam during the measurements (see Fig. S3 in Supplementary Information).

The temporal resolution was limited by the X-ray pulse duration of 100 ps, and the spatial resolution emerged from a convolution of the X-ray focal spot size and position jitter between the sample and X-ray beam. To achieve optimal spatial resolution, we ensured the projected X-ray footprint lied along the uniform direction of the 1D TG. The X-ray spatial scan direction then proceeded along the direction of optical intensity modulation, sampling the TG oscillations with the highest possible spatial resolution (see Fig. 1b).

In this work, different locations along the TG profile were measured by displacing the TG pattern relative to the fixed X-ray and crystal locations. Because we always probed the same sample location, we predominantly measured light-induced spatial inhomogeneity, distinguishing its effects from contributions from intrinsic spatial heterogeneities in the sample. Thus, “position” in our context refers to the position of the TG profile relative to the stationary X-ray probe.

Uncertainties for all reported results are either smaller than the markers used in each plot or indicated by error bars. For fixed *θ* measurements, the error bars show the standard errors of repeated measurements. For *θ*–2*θ* scans, we report error bars from the X-ray counting statistics, the square root of the total integrated X-ray counts within the region of interest of the pixel area detector. For results calculated from Bragg peak fits, such as phase fraction and thermal energy, the nonlinear peak fit estimates the 1σ standard error of and the correlation coefficients between the fit parameters. We then propagate this error to the final results, through the calculations presented in the Supplementary Information.

## Supplementary Information

Supplementary Information.

## Data Availability

The data that support the findings of this study are available from the corresponding author upon reasonable request.
