# Supplementary material for: Optical transient grating pumped X-ray diffraction microscopy for studying mesoscale structural dynamics
Source: Sci Rep. 2021 Sep 29;11:19322. doi: 10.1038/s41598-021-98741-y (PMC8481406; doi:10.1038/s41598-021-98741-y)
Supplement: Supplementary file 1 — Supplementary Information. [file 41598_2021_98741_MOESM1_ESM.pdf]

Supplementary Information for:

Optical transient grating pumped x-ray diffraction microscopy for studying mesoscale structural dynamics

Travis D. Frazer[1†], Yi Zhu[2†], Zhonghou Cai[2], Donald A. Walko[2], Carolina Adamo[3], Darrell G. Schlom[3,4,5], Eric E. Fullerton[6], Paul. G. Evans[7], Stephan O. Hruszkewycz[1], Yue Cao[1*], Haidan Wen[2*]

[1]Materials Science Division, Argonne National Laboratory, Lemont, IL 60439
[2]Advanced Photon Source, Argonne National Laboratory, Lemont, IL 60439
[3]Department of Materials Science and Engineering, Cornell University, Ithaca, NY 14853
[4]Kavli Institute at Cornell for Nanoscale Science, Ithaca, NY 14853
[5]Leibniz-Institut für Kristallzüchtung, Max-Born-Str. 2, 12489 Berlin, Germany
[6]Center for Memory and Recording Research, University of California San Diego, La Jolla, CA 92903
[7]Department of Materials Science and Engineering, University of Wisconsin−Madison, WI 53706

[†]equal contribution
*wen@anl.gov, yue.cao@anl.gov

Experimental setup:

We adapt a standard Michelson interferometer to construct our transient optical grating pump. One arm is slightly offset spatially to retain spatial separation between the two beams exiting the interferometer, as shown in Fig. S1. The spatially separated beams are focused by a f=30 cm (for BFO) or f=15 cm (for FeRh) lens with a crossing angle that varies as a function of the beam separation distance. A translation stage in one of the two beam paths enables synchronization between the two arms, which is necessary to interfere the two optical pulses at the sample. The lens is mounted on a piezo stage for scanning the TG pattern with respect to the x-ray probe and the sample. The 35 nm thick BFO film is grown on a $SrTiO_3$ (001) substrate by molecular-beam epitaxy. The 27 nm thick FeRh film is grown on an MgO (001) substrate by comagnetron sputtering.

The crossing angle, ξ, and the wavelength, λ, of the optical pump beams define the period of the TG excitation profile, as shown in Eqn. 1-2 of the main text. To precisely measure the TG period via local x-ray diffraction, we first calibrate the lens translation mechanism. Placing a camera with known pixel size at the sample plane, we measure how much each optical pump beam is displaced as a function of lens translation. This provides the real space TG relative position for x-ray diffraction measurements on each sample, which are recorded as a function of lens translation.

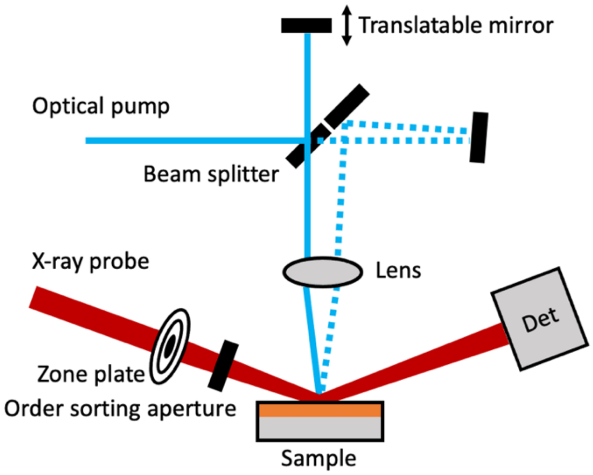

Figure S1: Experimental setup and optical interferometer. The 355 nm pump pulse enters the custom interferometer from the upper left. A 50-50 beam splitter reflects one arm (solid line) to a translatable retro-reflecting mirror. The other, transmitted arm (dotted line) reflects from a pair of mirrors aligned to achieve spatial separation between the two arms. The optical beams cross at the sample surface, aligned with the focused x-ray probe. The x-rays are focused by a zone plate and spatially filtered by an order sorting aperture. A pixelated detector collects the diffracted x-rays.

The x-ray beam is focused by a Fresnel zone plate, and an order sorting aperture selects only the first order focused photons to illuminate the sample. We characterize the x-ray beam size at the sample by scanning the sharp edge of a chromium film while recording the x-ray fluorescence intensity from Cr. Fitting an error function to the measured fluorescence as a function of x-ray position yields an x-ray full width at half maximum (FWHM) of 270 nm, as shown in Fig. S2. Repeated measurements show the focused beam does not damage the samples (see Fig. S3).

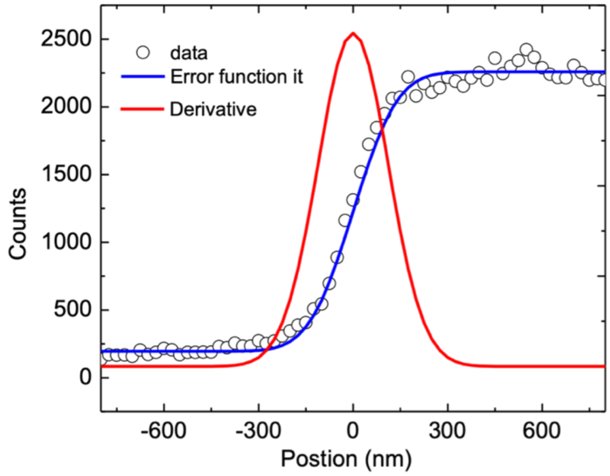

Figure S2: X-ray spot size measurement. We use a knife-edge test to determine the x-ray spot size. A 20 nm-thick chromium film provides the sharp edge. The x-ray fluorescence intensity of chromium is measured as the Cr edge is swept across the x-ray beam. Error function fitting indicates an x-ray FWHM of 270 nm.

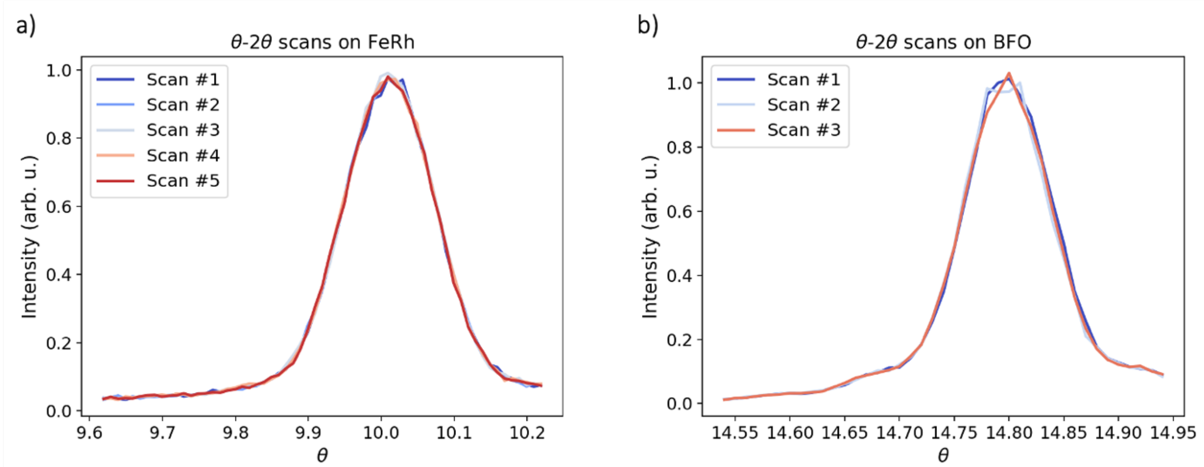

Figure S3: Damage tests for focused x-ray beam. Repeated θ-2θ scans in the same location on (a) FeRh and (b) BFO over the course of ~1 hour show no significant change to the Bragg peak shape. This indicates there is no x-ray induced sample damage during our measurements.

As described in Methods, a pixel area detector collects the diffracted x-ray beam that scatters from the sample. We use one region of interest around the main peak on the detector to integrate the total x-ray counts in the Bragg peak and a large region of interest to collect fluorescent scattering far away from the Bragg peak to serve as a beam intensity monitor. By symmetrically scanning the sample rotation (x-ray incident angle θ) and the detector arm (2θ), the x-ray intensity normalized by the intensity monitor defines the Bragg peak shape. As shown in Figs. 2-3 of the main text, the peak shape is best fit by a Voigt function, which convolves a Gaussian and a Lorentzian line shape to capture the contributions from the divergence of the zone-plate-focused x-ray beam and the finite film thickness. The zone plate we used had a 150 µm diameter and a 80 nm outer zone width, which gives a focal length of 117 mm and a divergence of 0.073° at 12 keV. The 27 nm FeRh film and 35 nm BFO film thicknesses estimate widths of 0.11° and 0.087°, respectively.

Strain calculation and biexponential fitting for BFO:

To convert the measured x-ray intensity at a fixed θ into strain in BFO, we first establish how the Bragg peak shape changes upon photoexcitation. As shown in Fig. 2(a) of the main text, θ-2θ scans at zero and maximum absorbed fluence quantify how the peak center, amplitude, and width all change. Assuming these parameters each change linearly between the two extremes, we calculate the x-ray intensity at the fixed θ position as a function of peak center. We find the intensity change is well fit by a straight line, which, once the peak center is converted into strain, provides the intensity-to-strain conversion quoted in the main text.

With our 100 ps temporal resolution, we resolve two characteristic decay times in photoexcited BFO – one on a nanosecond timescale and one on a microsecond timescale, captured by two

distinct data sets as shown in Fig. 2(d) of the main text. We then employ a sequential fitting procedure to find the biexponential function that fits both decays.

First, we use an unconstrained biexponential fit on the short timescale data to accurately determine the nanosecond decay constant. We then use this decay constant as a fixed input for the biexponential fit to the long timescale data, and thereby obtain the best-fit microsecond decay constant. We confirm this method of separately fitting each data set is appropriate by additionally testing the sensitivity of each fit to its respective disparate decay constant. For example, on the short timescale data, we test the sensitivity of the nanosecond decay constant fit to variations in the microsecond decay constant. We find that the short (long) timescale data reliably fit to the same short (long) decay constants, even when varying the disparate decay constant by a factor of 2.

Two-Voigt fits to FeRh rocking curves:

As shown in Fig. 4(b) in the main text, we fit two Voigt functions to the mixed phase Bragg peak of FeRh at each delay time and TG position. We first determine the individual peak shape by fitting single-phase Bragg peaks. This allows us to fix the Gaussian width defined by the zone plate, so that the Lorentzian width is the only fit width within the Voigt function for each phase, tracking the local heating. The other fit parameters are then the AFM and FM peak amplitudes, the AFM and FM peak centers, and a constant background.

To help the fit converge, we constrain the fit parameters. We find the AFM peak width and the background to be nearly constant across the dataset, and so constrain them to their average values for the final fit. The amplitudes of both peaks are constrained to be positive. The FM peak width must be within a reasonable range compared to single-peak fits, so as not to have an extremely wide peak behave like a second background offset. To constrain both the AFM and FM peak centers, we use the information from the fluence-dependent scans in Fig. 3(b) of the main text. The AFM peak can only vary between its measured positions at negative delay times (no excitation) and at the onset of the phase transition. The FM peak center cannot go beyond the minimum lattice spacing defined by extrapolating the linear fit in Fig. 3(b) to the onset of the phase transition near 1mJ/cm^2. Comparing the fully cooled AFM peak at late times in the TG valley from the Fig. 4 dataset to the measurement of the unexcited peak in the fluence-dependent dataset, we identify a small (+0.008°) drift in the theta motor between these two measurements near the beginning and end of the beamtime. We apply this small correction before using the values derived from the fluence-dependent data to constrain the fits for Fig. 4.

Thermal energy conversions and comparisons:

From the set of rocking curves on FeRh as a function of delay time and TG position, we calculate the thermal energy in the system using the measured Bragg peak amplitudes and peak positions together with the material properties listed in Table S1. First, we calculate the energy that goes into promoting FeRh across the phase transition as a function of TG position, *x*, and delay time, *t*. We use the AFM and FM peak amplitudes, A_{AFM} and A_{FM}, respectively, to define the FM phase fraction within the probed volume:

$$\Gamma_{FM}(x,t) = \frac{A_{FM}(x,t)}{A_{AFM}(x,t) + A_{FM}(x,t)}. \qquad S1$$

From the FM phase fraction, we calculate the energy density that goes into the phase transition

$$E_{trans}(x,t) = \Gamma_{FM}(x,t) * L * \rho * D, \qquad S2$$

where *L* is the latent heat of transition, and *D* is the film thickness (27 nm for FeRh). The density, ρ, is calculated from the standard atomic weights and the AFM lattice constant at the phase transition in Ref. 26 of the main text. Note that integrating over thickness has here been simplified to a factor of *D*, as the x-ray measurement naturally integrates over the full thickness of the film and does not resolve any variations in the depth direction.

Next, we calculate how much energy has gone into heating the FM phase, using the measured thermal expansion at all points. This starts with calculating the lattice parameter, *d*, of the FM phase, using the x-ray wavelength, λ_{x-ray}, and the measured peak position, θ:

$$d_{FM}(x,t) = \frac{\lambda_{x-ray}}{2\sin\left(\theta_{FM}(x,t)\right)}. \qquad S3$$

At long delay times in the TG valley, this calculation yields the minimum lattice parameter for the FM phase, d_{cooled} = 3.006 Å, where it has cooled back down to the transition temperature. Using this value to compute the percent change in the FM lattice parameter, we obtain the effective lattice temperature increase of the FM phase above the transition temperature at all points,

$$\Delta T_{FM}(x,t) = \left(\alpha_{FM}\frac{1+\nu}{1-\nu}\right)^{-1}\frac{d_{FM}(x,t) - d_{cooled}}{d_{cooled}}, \qquad S4$$

where α_{FM} and ν are the linear coefficient of thermal expansion and Poisson's ratio, respectively, for the FM phase. The maximum temperature increase according to this approach is 550 K, giving an absolute FM temperature of 920 K at the TG peak at time 0.

From this change in temperature, we then calculate the energy density that goes into heating the FM phase that is present,

$$E_{FM}(x,t) = \Delta T_{FM}(x,t) * \Gamma_{FM}(x,t) * C_p * \rho * D, \qquad S5$$

where C_p is the specific heat of the FM phase. Note that both E_{trans} and E_{FM} have units of [mJ/cm^2], identical to the units of optical fluence. This enables easy comparison to the independently characterized optical pump parameters.

Property	Value	Reference
Lattice constant, FM (Å)	2.9962	Ref. 26 in main text
Lattice constant, AFM (Å)	2.9878	Ref. 26 in main text
Poisson's ratio, FM	0.25	Ref. 30 in main text
Poisson's ratio, AFM	0.32	Ref. 30 in main text
Linear CTE, for FM (1/K)	$6.7x10^{-6}$	Ref. 26 in main text
Linear CTE, for AFM (1/K)	$9.5x10^{-6}$	Ref. 26 in main text
Latent heat of transition (kJ/kg)	3.4	Ref. 30 in main text
Specific heat, C_P, for FM (J/kg/K)	400	Ref. 29 in main text
Specific heat, C_P, for AFM (J/kg/K)	325	Ref. 29 in main text
Calculated density (kg/m^3)	9880.2	Atomic weights and Ref. 26

Table S1: Literature values for FeRh material properties. CTE is the coefficient of thermal expansion. The calculated density uses the AFM lattice constant to define the unit cell volume, and the standard atomic weights of Fe and Rh to define the mass.

To check our energy conversions, we compare the energy densities, E_{trans} and E_{FM}, derived from x-ray measurements to the optical fluence absorbed by the sample. For the TG position at the peak intensity and the delay time of 0 ns (TG and x-ray coincident arrival time), we calculate $E_{trans} =$ 0.080 mJ/cm^2 and $E_{FM} =$ 5.1 mJ/cm^2. Using the heat capacity of the AFM phase, we also add 0.69 mJ/cm^2 for the energy required to heat FeRh from 295 K to the phase transition at 375 K. Adding these three components, we calculate a total energy density of 5.9 mJ/cm^2 required for the structural change measured by x-ray diffraction at the TG peak.

To compare with the supplied energy density by the laser excitation, we first measure the optical power at the sample position in one of the two TG arms, which is 77.85 mW at 54 kHz, with a 190x265 μm^2 average elliptical spot size (average between the two arms' slightly varying spot sizes). Therefore, the incident fluence in one arm is F_1 = 3.65 mJ/cm^2, and the incident fluence at the peak of the TG is F_{inc} = 4*F_1 (4x enhancement at TG peak compared to single-arm fluence). The absorbed fluence is then given by F = (1-R)(1-A)*F_{inc}, where R is the reflectivity of FeRh, and A is the fraction of incident light transmitted through the thin film. From Ref. 31 in the main text, we can estimate the real and imaginary parts of the dielectric function of FeRh at 3.5eV to be $\varepsilon_1 = -7$ and $\varepsilon_2 = 8$. We then calculate R = 0.6 using

$$R = \frac{(n_1 - 1)^2 + n_2^2}{(n_1 + 1)^2 + n_2^2}, \qquad S6$$

$$\varepsilon_1 = n_1^2 - n_2^2, \qquad S7$$

$$\varepsilon_2 = 2n_1 n_2, \qquad S8$$

where n_1 and n_2 are the real and imaginary parts of the refractive index. We next calculate that A = 0.058 using

$$A = e^{-\alpha * D}, \qquad S9$$

$$\alpha = {}^{4\pi n_2}/_{\lambda}, \qquad S10$$

where D is the film thickness, α is the absorption coefficient, and λ is the optical pump wavelength. Using R = 0.6 and A = 0.058, we calculate an estimated absorbed fluence at the TG peak of 5 mJ/cm^2, as quoted in the main text.

Optical penetration depths in FeRh, BFO, and STO:

From the above calculation of α, we can determine the optical penetration depth of 355 nm light in FeRh as $1/\alpha = 9.5$ nm. With the reflectivity of R=0.6, this means only (1-R)*A = 2% of the incident light penetrates through the 27 nm FeRh film.

For BFO, the optical absorption coefficient for 355 nm light is $\alpha = 3.558 \times 10^5$ cm^{-1}, and R=0.32, from Ref. 32 in the main text. This provides an optical penetration depth of 28 nm, compared to the 35 nm thickness of the film. All together, this predicts 20% of incident pump light reaches the STO substrate through the BFO film, neglecting reflection from the BFO/STO interface.

In STO, the optical penetration depth is 295 nm, from $\alpha = 0.33937 \times 10^5$ cm^{-1}, using Ref. 33 in the main text. Thus, pump light is also absorbed in the substrate, albeit less strongly than in the BFO and FeRh films. We neglect this effect in the case of FeRh because so little of the incident light reaches the transparent MgO substrate. In the case of BFO, we expect any substrate pumping effects are negligible in our results because the estimated temperature rise in STO is much less than that in BFO. Given the two materials' similar heat capacities, the ~10× longer optical penetration depth in STO would lead to a 10× lower temperature rise, compared to BFO. Combined with the fact that only 20% of the incident light reaches the STO substrate, neglecting reflection from the BFO/STO interface, the temperature rise due to optically pumping the STO substrate should be ~2% of the temperature rise in BFO.
